# Increased alpha-9 human papillomavirus species viral load in human immunodeficiency virus positive women

**DOI:** 10.1186/1471-2334-14-51

**Published:** 2014-01-31

**Authors:** Zizipho ZA Mbulawa, Leigh F Johnson, Dianne J Marais, Inger Gustavsson, Jennifer R Moodley, David Coetzee, Ulf Gyllensten, Anna-Lise Williamson

**Affiliations:** 1 Institute of Infectious Disease and Molecular Medicine, University of Cape Town, Anzio Road, Observatory, 7925 Cape Town, South Africa; 2 Centre for Infectious Disease Epidemiology and Research, School of Public Health and Family Medicine, University of Cape Town, Cape Town, South Africa; 3 School of Public Health and Family Medicine, Women’s Health Research Unit, University of Cape Town, Cape Town, South Africa; 4 Department of Immunology, Genetics and Pathology, Rudbeck Laboratory, University of Uppsala, S-751 85 Uppsala, Sweden; 5 National Health Laboratory Service, Groote Schuur Hospital, Observatory, Cape Town 7925, South Africa; 6 Center for HIV and STIs, National Institute for Communicable Disease, National Health Laboratory Service, Groote Schuur Hospital, Observatory, Cape Town 7925, South Africa

**Keywords:** Human papillomavirus, Human immunodeficiency virus, Viral load

## Abstract

**Background:**

Persistent high-risk (HR) human papillomavirus (HPV) infection and increased HR-HPV viral load are associated with the development of cancer. This study investigated the effect of human immunodeficiency virus (HIV) co-infection, HIV viral load and CD4 count on the HR-HPV viral load; and also investigated the predictors of cervical abnormalities.

**Methods:**

Participants were 292 HIV-negative and 258 HIV-positive women. HR-HPV viral loads in cervical cells were determined by the real-time polymerase chain reaction.

**Results:**

HIV-positive women had a significantly higher viral load for combined alpha-9 HPV species compared to HIV-negative women (median 3.9 copies per cell compared to 0.63 copies per cell, P = 0.022). This was not observed for individual HPV types. HIV-positive women with CD4 counts >350/μl had significantly lower viral loads for alpha-7 HPV species (median 0.12 copies per cell) than HIV-positive women with CD4 ≤350/μl (median 1.52 copies per cell, P = 0.008), but low CD4 count was not significantly associated with increased viral load for other HPV species. High viral loads for alpha-6, alpha-7 and alpha-9 HPV species were significant predictors of abnormal cytology in women.

**Conclusion:**

HIV co-infection significantly increased the combined alpha-9 HPV viral load in women but not viral loads for individual HPV types. High HR-HPV viral load was associated with cervical abnormal cytology.

## Background

Persistent cervical infection with high-risk (HR) human papillomavirus (HPV) leads to the development of cervical lesions and this is accelerated in human immunodeficiency virus (HIV)-positive women [1-3]. HIV co-infected women appear to progress to cervical cancer much earlier than HIV-negative women [4,5]. Immune competence plays an important role in cervical disease progression. Women with low CD4 cell counts have a higher HPV viral load, and a greater risk of persistent HPV infection and cervical cancer [6,7].

The increased HPV viral load and low CD4 counts among HIV-positive women increases the probability of developing cervical cancer [7-9]. The HPV viral load is positively associated with the extent of cervical abnormalities. The HPV viral load is also positively associated with the frequency of HPV DNA integration [10,11]. However it is not clear whether HIV has an effect on cervical abnormalities after controlling for known associations with HR-HPV viral load.

In the present study we detected and quantified genital HPV types in women using the in-house real-time polymerase chain reaction (RT-PCR). The objectives of the study were (i) to detect and quantify the respective HR-HPV types in HIV-positive and HIV-negative women; (ii) to investigate the effect of HIV co-infection on HR-HPV viral load; and (iii) to investigate predictors of cervical abnormality.

## Methods

### Study population and specimen collection

Study participants were recruited through clinics, bus stops and taxi ranks. Eligible individuals were asked to come to Manyanani clinic (a research clinic based in Empilisweni centre, Gugulethu, Cape Town, South Africa) with their sexual partner between 2006 and 2009. The Research Ethics Committee of the University of Cape Town approved all aspects of the investigation (reference: 258/2006) and informed consent was obtained. A total of 258 HIV-positive women and 292 HIV-negative women were enrolled for this study. For enrolment we did not select certain participants; however, as they were coming in they were enrolled [12]. Samples were collected and stored as described by Mbulawa et al., [13].

### Detection and quantification of HPV DNA

DNA was extracted from cervical cells using the MagNA Pure Compact Nucleic Acid Isolation Kit (Roche diagnostics, Mannheim, Germany) and automated Roche MagNA Pure Compact machine. DNA was stored at -20°C and shipped to University of Uppsala, Sweden for detection and quantification of HR-HPV DNA. The real-time PCR assay used in this study has been reported to have similar sensitivity and specificity to Hybrid Capture 2 (Qiagen, Hilden, Germany), a United States Food and Drug Administration approved assay [14]. HR-HPV was detected and quantified as described by Gustavsson et al., [14].

The assay used was based on four parallel real-time PCRs from each DNA sample, one reaction to quantify the amount of a human single-copy gene (house-keeping gene; Homo sapiens hydroxymethylbilane synthase (HMBS); GenBank accession no. M95623.1) and the three other reactions to detect and quantify HR-HPVs (HPV-16, -18, -31, -33, -35, -39, -45, -51, -52, -56, -58, and -59). The results are presented as individual types, except for HPV-18 and -45, which are detected as a phylogenetically-related group, and HPV-33, -52, and -58, which are similarly detected. The real-time PCR assay was carried-out in a final volume of 25 μl, containing 3 μl template DNA from cervical cells, Taqman® Universal PCR master mix with no AmpErase® UNG (Applied Biosystems, Inc, Foster City, CA, USA), 3.1 μg of bovine serum albumin (Sigma, St Louis, MO, USA), 200 nM of each primer (Thermo Hybaid, Waltham, MA, USA) and probe (Applied Biosystems, Inc, Foster City, CA, USA). Primers and probes are listed in Table 1 of Gustavsson et al., [14].

**Table 1:** HR-HPV viral load per cell in women according to HIV-status

**HPV type**	**HIV-negative women**		**HIV-positive women**		**P-value**
	n*	**Median (IQR)**	n*	**Median (IQR)**	
HPV-16	8	0.43 (0.04–1.12)	29	2.35 (0.12–17.6)	0.238
HPV-18/45	9	0.27 (0.01–4.41)	41	1.33 (0.10–18.8)	0.398
HPV-31	9	0.25 (0.04–4.88)	15	0.70 (0.01–1.47)	0.698
HPV-33/52/58	22	1.02 (0.09–17.1)	67	3.18 (0.17–105)	0.220
HPV-35	16	0.51(0.03–4.78)	24	1.14 (0.08–10.1)	0.456
HPV-39	7	0.10 (0.00–44.0)	14	0.36 (0.00–6.93)	0.709
HPV-51	3	1.15 (0.01–3.90)	19	2.17 (0.33–16.1)	0.363
HPV-56	9	0.02 (0.01–0.33)	25	0.09 (0.01–13.9)	0.339
HPV-59	8	0.09 (0.00–1.21)	14	0.01 (0.00–4.90)	0.838
α5 HPV species	3	1.15 (0.01–3.90)	19	2.17 (0.33–16.1)	0.363
α6 HPV species	9	0.02 (0.01–0.33)	25	0.09 (0.01–13.9)	0.339
α7 HPV species	24	0.15 (0.01–5.88)	58	0.65 (0.05–11.0)	0.245
α9 HPV species	47	0.63 (0.08–7.72)	100	3.90 (0.22–53.2)	**0.022**

**Note:α5 and α6** HPV species include HPV-51 and -56 respectively; **α7** HPV species includes HPV-18, -39, -45 and -59; **α9** HPV species includes HPV-16, -31, -33, -35, -52 and -58. P-values were calculated by Mann-Whitney test (two-tailed). Significant P-values are in bold.

Amplification and detection steps were performed using the 7900 HT Sequence Detection System (Applied Biosystems, Inc, Foster City, CA, USA). The amplification ramp includes an initial hold program of 10 minutes at 95°C followed by a two-step cycle consisting of 95°C for 15 seconds and 57°C for 1 minute that was repeated 40 times. The sensitivity of the HPV assay was determined using plasmids containing the full genome of different HPV types according to Moberg et al. [13] and Gustavsson et al. [15] Standard curves ranging from 10^2^ to 10^5^ copies were established for each HPV type or group of HPV types to be detected Gustavsson et al. [16] A highly significant linear relationship was seen between HPV copy number and threshold cycle (Ct) for all HPV types detected by the system. The threshold for a positive HPV type was set at 10 copies per PCR. Similarly, a linear relationship was seen between copy number of the human HMBS gene and threshold cycle, and as threshold for inclusion in the study a copy number of 10 genomic equivalents were used. The RT-PCR data was analysed with the applying software SDS version 2.2. HPV copies per cell were calculated by dividing HPV copies per sample by copies of house-keeping gene HMBS.

### Statistical analyses

HPV prevalence differences were evaluated using the *χ*^2^ test (EpiInfo Version 5 Statcalc). Mann-Whitney tests were used when analysing the effect of HIV status and CD4 counts on HR-HPV viral load. Univariate and multivariate logistic regressions were conducted to assess predictors of abnormal cytology, using STATA 11.0 (StataCorp, College Station, TX, USA). In all analyses P-values ≤0.05 were considered significant. When calculating viral loads for groups of HPV types, the viral loads of individual types were added together in people who were infected with more than one of the types in the group.

## Results

### HR-HPV prevalence in women according to HIV status

**Figure 1:**
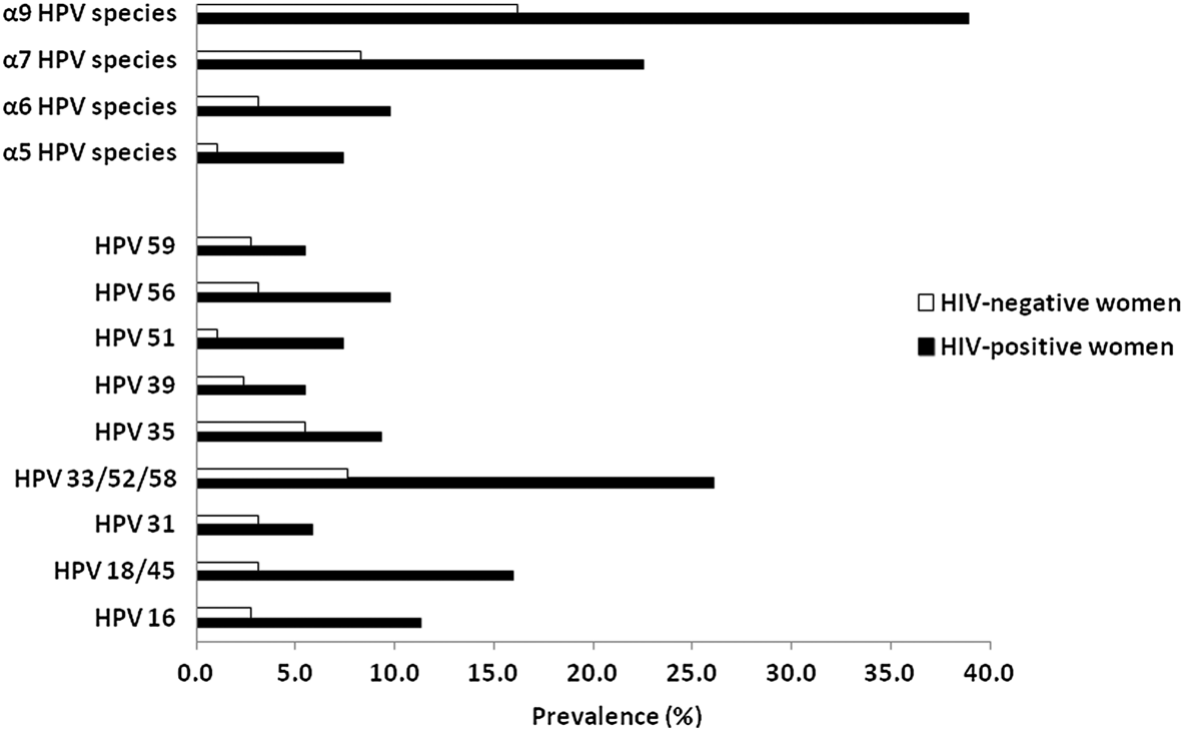
**High-risk human papillomavirus genotypes in women according to HIV status.** White bars indicate HIV-negative women and black bars indicate HIV-positive women.

**Figure 2:**
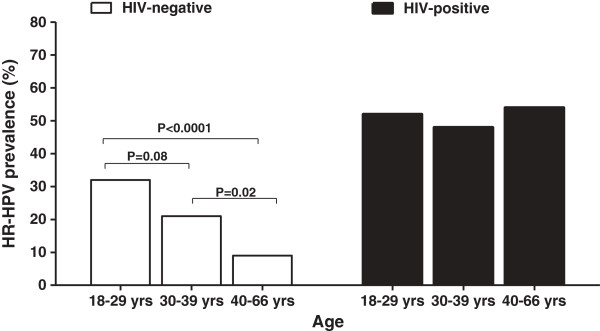
**High-risk human papillomavirus prevalence in women according to HIV status and age.** White bars indicate HIV-negative women and black bars indicate HIV-positive women.

To determine if the genital sampling was adequate, the house keeping gene Homo sapiens hydroxymethylbilane synthase (HMBS) was quantified in each specimen. HMBS copies were found to be <10 in 3/550 (0.5%) cervical samples. The samples with <10 HMBS copies were not included in the analysis. HIV-positive women had a significantly higher prevalence of HR-HPV compared to HIV-negative women (51% 131/257; 21% 61/290, respectively, P < 0.0001). Among the HR-HPV positive participants, HIV-positive women were found to have significantly higher prevalence of multiple HR-HPV infections compared to HIV-negative women (49% 65/131; 28% 17/61, respectively, P = 0.001). HIV-positive women were found to have a higher prevalence of all the HR-HPV types at both individual and species level compared to HIV-negative women (Figure 1). The mean ages of HIV-positive and HIV-negative women were 34 years (range, 18-65 years) and 35 years (range, 18-66 years) respectively. HR-HPV prevalence was significantly higher in women aged 18-29 years (32%, 35/108) compared to women aged 40-66 years (9%, 9/101, P = <0.0001) but not to women aged 30-39 years (21%, 17/81, P = 0.08, Figure 2).

### HR-HPV viral load

Median HR-HPV viral loads differed substantially between types, ranging from 0.018 for type 59 (IQR: 0.005-0.82) to 2.02 for type 51 (IQR: 0.30-14.3) and 2.73 for types 33, 52 and 58 combined (IQR: 0.16-73.2). The median HPV viral loads per cell were consistently higher in HIV-positive women compared to HIV-negative women (except for HPV-59) at the individual level; however, the differences were not statistically significant. When HPV types were grouped according to HPV species, HIV-positive women were found to have a significantly higher number of copies per cell for the α9 HPV species, compared to HIV-negative women (median 3.90 compared to 0.63 copies per cell, P = 0.022) but not for α7 HPV species (Table 1). HIV-positive women with CD4 counts >350/μl had significantly lower α7 HPV species viral loads (N = 26, median 0.12 copies per cell) than HIV-positive women with CD4 ≤350/μl (N = 32, median 1.52 copies per cell, P = 0.008), but none of the median HPV viral loads for other HPV types or species were found to be significantly lower in HIV-positive women with CD4 counts >350/μl (results not shown). Further analysis suggested that species 9 viral loads, and viral loads for types 33, 52 and 58 combined, were significantly higher in women with CD4 counts >500 than in lower CD4 strata, while species 7 viral load levels were highest in the 201-350 category (Additional file 1: Table S1). The associations between HPV viral load and age was investigated and it was found to be significant only in the case of HPV-39; for each year of increase in age, there was a 0.079 (95% confidence interval (CI): 0.01-0.15) reduction in the logarithm of the HPV-39 viral load.

### Predictors of abnormal cervical cytology

A total of 388 (72%) had normal cervical cytology, 52 (10%) had atypical squamous cell of undetermined significance (ASCUS), 82 (15%) had low-grade squamous intraepithelial lesion (LSIL), and 12 (2%) had high-grade squamous intraepithelial lesion (HSIL). Table 2 presents factors that were associated with abnormal cervical cytology (univariate analysis). HIV-positive women were at greater risk of having abnormal cervical cytology [odds ratio (OR), 2.35 (95% CI: 1.59-3.48)]. HIV-positive women with CD4 counts <350/μl were not found to have higher risk of having abnormal cervical cytology compared to those with CD4 counts ≥350/μl [OR, 1.42 (95% CI: 0.85-2.38)]. The HIV viral load was also not found to be significantly associated with abnormal cervical cytology [OR, 1.03 (95% CI: 0.84-1.26) per unit increase in log viral load].

**Table 2:** The predictors of abnormal cervical cytology in women (univariate analysis)

**Variable**	**OR**	**95% CI**	**P-value**
Age group			
<30 years	1		0.070
30–39 years	1.31	0.84–2.03	
≥40 years	0.73	0.44–1.20	
HIV-positive	2.35	1.59–3.48	**<0.001**
Living together with study partner	0.93	0.63–1.37	0.700
Per 1-year increase in relationship duration	0.92	0.84–1.01	0.065
Per 1-year increase in age at first sex	1.07	0.99–1.16	0.086
Per 10-unit increase in number of sex acts/month	1.01	0.87–1.16	0.930
Lifetime number of sexual partners			
1–2	1		0.500
3–5	0.79	0.52–1.19	
>5	0.83	0.46–1.50	
Ever used a condom with current study partner	1.23	0.81–1.87	0.340
Experienced genital discharge in last 6 months	1.09	0.66–1.81	0.730
Experienced genital ulcer in last 6 months	1.50	0.7–3.20	0.310
α5 HPV infection	3.40	1.44–8.06	**0.006**
α6 HPV infection	5.59	2.69–11.62	**<0.001**
Species 7 HPV infection			
None	1		**<0.001**
Species 7 VL = 0.6 copies per cell (median)	4.82	2.82–8.24	
Per unit increase in log copies per cell	1.55	1.15–2.08	
Species 9 HPV infection			**<0.001**
None	1		
Species 9 VL = 2.5 copies per cell (median)	7.23	4.58–11.40	
Per unit increase in log copies per cell	1.66	1.28–2.15	

Note: *Covariate data were missing for some women and the total observations therefore sum to less than 550 for certain univariate analyses. VL: viral load per cell, **α5** HPV species is HPV-51; **α6** HPV species is HPV-56; **α7** HPV species are HPV-18, -39, -45 and -59; **α9** HPV species are HPV-16, -31, -33, -35, -52 and -58.

Other predictors of abnormal cervical cytology were found to be infection with α5, α6, α7 or α9 HPV species. In women infected with α7 HPV species, the α7 HPV viral load was strongly associated with abnormal cervical cytology [OR 1.55 (95% CI: 1.15-2.08) per unit increase in log viral load]. Similarly, in women infected with α9 HPV species, the odds of abnormal cervical cytology were significantly associated with α9 HPV viral load [OR 1.66 (95% CI: 1.28-2.15) per unit increase in log viral load] (Table 2). In multivariate analysis HIV status was not a significant predictor of abnormal cytology, and only high α6, α7 and α9 HPV species viral loads remained as predictors of abnormal cytology (Table 3).

**Table 3:** The predictors of abnormal cervical cytology in women, (multivariate analysis)

**Variable**	**OR**	**95% CI**	**P-value**
**Species 6 HPV infection**	5.04	2.15-12.3	**<0.001**
**Species 7 HPV infection**			
None	1		**<0.001**
Species 7 VL = 0.6 copies per cell (median)	3.11	1.70–5.71	
Per unit increase in log copies per cell	1.67	1.18–2.36	
**Species 9 HPV infection**			**<0.001**
None	1		
Species 9 VL = 2.5 copies per cell (median)	6.19	3.81–10.10	
Per unit increase in log copies per cell	1.62	1.24–2.12	

**Note:** VL: viral load per cell, **α6** HPV species is HPV-56; **α7** HPV species are HPV-18, -39, -45 and -59; **α9** HPV species are HPV-16, -31, -33, -35, -52 and -58.

## Discussion

In this study we investigated the HR-HPV viral load in HIV-positive and HIV-negative women. To our knowledge this is the first study to report individual HR-HPV type viral loads in HIV-positive and HIV-negative women detected by this kind of RT-PCR assay. HIV co-infection significantly increased cervical HR-HPV prevalence in HIV-positive women. The prevalence of HR-HPV in HIV-positive women (73.3%) and HIV-negative women (45.5%) was lower than that reported by Luchters et al., [17]. This difference could be due to the fact that the current study included 12 HR-HPV types, while that of Luchters et al. studied 15 types , 3 more HR-HPV types (HPV-53, -66 and -68) compared to our study [17]. The high HR-HPV prevalence and viral load in HIV-positive women may be due to the immune suppression caused by HIV infection, which could also result in the reactivation of latent infection and a high susceptibility to new HPV acquisition [6,18]. The increased rate of latent HPV infection reactivation and a high susceptibility to new HPV acquisition among HIV-positive individuals also results in a high prevalence of multiple infections [6,19].

HIV-positive women were found to have increased α9 HPV viral load compared to HIV-negative women, similar to findings reported in other studies [2,15,17,19,20]. The observed significantly higher viral load in HIV-positive women when α9 HPV species were combined but not for the individual HPV types may be explained by two factors. Firstly, the larger numbers of α9 HPV-positive cases, when compared to the numbers positive for individual types, implies greater statistical power to detect an HIV effect. Secondly, HIV is known to increase the risk of multiple HPV types [19], therefore an individual infected with multiple α9 types is likely to have a higher combined α9 viral load than an individual infected with a single α9 type.

The association of the immune system with HPV viral load is reflected in the increasing α7 HPV viral load with decreasing CD4 count among HIV-positive women. Levi et al. reported that women with low CD4 counts were at increased risk of high HPV viral load and cervical abnormal cytology compared to women with higher CD4 counts [6]. We did not find a consistent effect of CD4 count on HPV viral load, similar to other studies demonstrating inconsistent effects of CD4 count on HPV viral load [7,16,21]. The low numbers of participants in the groupings for CD4 count limited the statistical power in determining the association between CD4 count and HR-HPV viral load.

HIV infection and high HPV viral load were found to be predictors of abnormal cytology, however; in multivariate analysis after controlling for HPV viral load, the effect of HIV status on this association was not statistically significant [22-24]. Infection with HPV species α6, α7 and α9 (and high viral loads in the case of species α7 and α9) were found to be predictors of abnormal cytology in this report. High HPV viral load has previously been reported as a predictor of HPV persistent infection and risk of developing precancerous lesions and cancer [25,26]. In the study of Fontaine et al. women who did not develop cervical abnormalities during follow-up were those with stable HPV viral load, while those who developed cervical disease were more likely to be those with increased HPV viral load [7].

## Conclusion

Although the current study is a cross-sectional study, we observed that HIV co-infection significantly influenced HR-HPV prevalence in women and α9 HPV viral load in women. High HR-HPV viral loads were found to be associated with cervical abnormal cytology. Data from this study will add to the growing HPV viral load information in South African HIV-positive and HIV-negative women.

### Ethical approval

Ethical approval was given by the Research Ethics Committee of the University of Cape Town (reference: 258/2006) and informed consent was obtained.

Some of the results from this manuscript were presented at the 25^th^ International Papillomavirus Conference and Clinical Workshop (Malmö, Sweden).

## Abbreviations

ASCUS: Atypical squamous cell of undetermined significance; CI: Confidence interval; HIV: Human immunodeficiency virus; HMBS: Hydroxymethylbilane synthase; HPV: Human papillomavirus; HSIL: High-grade squamous intraepithelial lesion; HR: High-risk; LSIL: Low-grade squamous intraepithelial lesion; RT-PCR: Real time polymerase chain reaction.

## Competing interests

The authors declare that they have no competing interests.

## Authors’ contributions

DC, DJM and ALW were responsible for study set-up and specimen collection. ZZAM was responsible for specimen storage. IG, UG and ZZAM were responsible for generating HPV viral load data. The first draft was written by ZZAM, with contributions from DJM, IG, UG, LFJ, DC, and ALW. Statistical analysis was conducted by LFJ. All authors were actively involved in the interpretation of the data, creation and revision of the manuscript; and approval of the final manuscript.

## Pre-publication history

The pre-publication history for this paper can be accessed here:

http://www.biomedcentral.com/1471-2334/14/51/prepub

## Supplementary Material

Additional file 1: Table S1 Differences in HPV viral load in HIV-infected women, by CD4 count. Click here for file
