# Supplementary material for: Increased alpha-9 human papillomavirus species viral load in human immunodeficiency virus positive women
Source: BMC Infect Dis. 2014 Jan 31;14:51. doi: 10.1186/1471-2334-14-51 (PMC3922074; doi:10.1186/1471-2334-14-51)
Supplement: Additional file 1: Table S1 — Differences in HPV viral load in HIV-infected women, by CD4 count. [file 1471-2334-14-51-S1.doc]

Supplementary table: Differences in HPV viral load in HIV-infected women, by CD4 count

|  | CD4 >500 | | CD4 351-500 | | CD4 201-350 | | CD4 ≤200 | |  |
| --- | --- | --- | --- | --- | --- | --- | --- | --- | --- |
|  | n | Median | n | Median | n | Median | n | Median | P^*^ |
| HPV-16 | 4 | 10.1 | 8 | 5.7 | 13 | 1.1 | 4 | 2.1 | 0.646 |
| HPV-18/45 | 9 | 0.09 | 8 | 0.33 | 17 | 2.57 | 7 | 0.22 | 0.739 |
| HPV-31 | 2 | 113 | 2 | 0.4 | 7 | 0.1 | 4 | 0.8 | 0.251 |
| HPV-33/52/58 | 14 | 31.9 | 15 | 0.25 | 25 | 3.2 | 13 | 3.7 | **0.018** |
| HPV-35 | 1 | 0.03 | 6 | 6.3 | 12 | 0.73 | 5 | 0.22 | 0.601 |
| HPV-39 | 1 | 0.009 | 5 | 0.001 | 4 | 1.296 | 4 | 2.234 | 0.187 |
| HPV-51 | 3 | 3.7 | 5 | 1.1 | 8 | 1.9 | 3 | 6 | 0.133 |
| HPV-56 | 8 | 0.04 | 4 | 3.42 | 11 | 0.24 | 2 | 141.7 | 0.383 |
| HPV-59 | 5 | 0.023 | 2 | 0.003 | 3 | 31.1 | 4 | 0.009 | 0.469 |
| α5 HPV species | 3 | 3.7 | 5 | 1.1 | 8 | 1.9 | 3 | 6.0 | 0.133 |
| α6 HPV species | 8 | 0.04 | 4 | 3.42 | 11 | 0.24 | 2 | 141.7 | 0.383 |
| α7 HPV species | 12 | 0.07 | 14 | 0.18 | 21 | 2.03 | 11 | 0.38 | **0.021** |
| α9 HPV species | 18 | 29.97 | 23 | 1.08 | 38 | 3.31 | 21 | 3.66 | **0.049** |

* Calculated using the chi-squared test for equality-of-medians. Highlighted values represent statistically significant results.
